# Assessment of plasma chitotriosidase activity, CCL18/PARC concentration and NP-C suspicion index in the diagnosis of Niemann-Pick disease type C: a prospective observational study

**DOI:** 10.1186/s12967-017-1146-3

**Published:** 2017-02-21

**Authors:** Isabel De Castro-Orós, Pilar Irún, Jorge Javier Cebolla, Victor Rodriguez-Sureda, Miguel Mallén, María Jesús Pueyo, Pilar Mozas, Carmen Dominguez, Miguel Pocoví, on behalf of the Spanish NP-C Group

**Affiliations:** 1 Department of Biochemistry and Molecular and Cellular Biology, Faculty of Science, University of Zaragoza, C. Pedro Cerbuna 12, 50009 Saragossa, Spain; 2 Instituto de Investigación Sanitaria Aragón (IIS Aragón), Saragossa, Spain; 3 Centro de Investigación Biomédica en Red (CIBERER), Instituto de Salud Carlos III, Saragossa, Spain; 4 Spanish Foundation for the Study and Therapy of Gaucher Disease, Saragossa, Spain; 5 Biochemistry and Molecular Biology Research Centre for Nanomedicine, Vall d’Hebron University Hospital, Barcelona, Spain

**Keywords:** Niemann-Pick disease type C, Chitotriosidase, CCL18/PARC, NP-C suspicion index, 7-ketocholesterol, Diagnosis, Screening

## Abstract

**Background:**

Niemann-Pick disease type C (NP-C) is a rare, autosomal recessive neurodegenerative disease caused by mutations in either the NPC1 or NPC2 genes. The diagnosis of NP-C remains challenging due to the non-specific, heterogeneous nature of signs/symptoms. This study assessed the utility of plasma chitotriosidase (ChT) and Chemokine (C–C motif) ligand 18 (CCL18)/pulmonary and activation-regulated chemokine (PARC) in conjunction with the NP-C suspicion index (NP-C SI) for guiding confirmatory laboratory testing in patients with suspected NP-C.

**Methods:**

In a prospective observational cohort study, incorporating a retrospective determination of NP-C SI scores, two different diagnostic approaches were applied in two separate groups of unrelated patients from 51 Spanish medical centers (n = 118 in both groups). From Jan 2010 to Apr 2012 (Period 1), patients with ≥2 clinical signs/symptoms of NP-C were considered ‘suspected NP-C’ cases, and NPC1/NPC2 sequencing, plasma chitotriosidase (ChT), CCL18/PARC and sphingomyelinase levels were assessed. Based on findings in Period 1, plasma ChT and CCL18/PARC, and NP-C SI prediction scores were determined in a second group of patients between May 2012 and Apr 2014 (Period 2), and NPC1 and NPC2 were sequenced only in those with elevated ChT and/or elevated CCL18/PARC and/or NP-C SI ≥70. Filipin staining and 7-ketocholesterol (7-KC) measurements were performed in all patients with NP-C gene mutations, where possible.

**Results:**

In total across Periods 1 and 2, 10/236 (4%) patients had a confirmed diagnosis o NP-C based on gene sequencing (5/118 [4.2%] in each Period): all of these patients had two causal NPC1 mutations. Single mutant NPC1 alleles were detected in 8/236 (3%) patients, overall. Positive filipin staining results comprised three classical and five variant biochemical phenotypes. No NPC2 mutations were detected. All patients with NPC1 mutations had high ChT activity, high CCL18/PARC concentrations and/or NP-C SI scores ≥70. Plasma 7-KC was higher than control cut-off values in all patients with two NPC1 mutations, and in the majority of patients with single mutations. Family studies identified three further NP-C patients.

**Conclusion:**

This approach may be very useful for laboratories that do not have mass spectrometry facilities and therefore, they cannot use other NP-C biomarkers for diagnosis.

**Electronic supplementary material:**

The online version of this article (doi:10.1186/s12967-017-1146-3) contains supplementary material, which is available to authorized users.

## Background

Niemann-Pick disease type C (NP-C) is a rare inherited lysosomal storage disorder with an estimated incidence of 1:120,000 live births [1]. Mutations in either of the two genes, *NPC1* or *NPC2*, have been described as the cause of the disease [1]. Approximately 95% of patients with a genetic diagnosis have *NPC1* mutations: *NPC1* encodes a large membrane glycoprotein with mostly late-endosomal localization [2]. Other patients have mutations in the *NPC2* gene, which encodes a small soluble lysosomal protein that binds cholesterol with high affinity [1, 3].

The diagnosis of NP-C remains challenging as neurological signs of the disease are extremely varied in terms of severity and age at onset [1, 4]. In particular, the age at onset of neurological manifestations has a major influence on disease progression and prognosis, and patients can be categorized on the basis of early-infantile, late-infantile, juvenile and adolescent/adult neurological onset to aid clinical management and family counselling [4]. Generally, patients with very early-onset disease are detected and diagnosed based on pronounced visceral symptoms such as prolonged neonatal jaundice, fetal hydrops and/or ascites [1, 5]. Diagnoses in later-onset patients depend more on the recognition of typical neurological signs such as vertical supranuclear gaze palsy (VSGP), developmental delay, cerebellar ataxia, and gelastic cataplexy, which may or may not be detected alongside splenomegaly. However, visceral symptoms can appear long before neurological signs and often go unrecognized, particularly in those with adolescent/adult-onset disease [1, 4].

The NP-C suspicion index (NP-C SI) was developed by an international team of experts for the detection of NP-C among patients suspected of having the disease, and is based on easily assessed patient clinical symptoms and medical history [6]. Patients scoring ≥70 on the NP-C SI should be considered as possibly having NP-C and should undergo further, specific laboratory tests. Scores <40 on the NP-C SI indicate a low likelihood of NP-C [4].

Niemann-Pick disease type C diagnoses can only be confirmed using specific laboratory tests [4]. Filipin staining in patient skin fibroblast cultures is a sensitive and specific test to identify impaired intracellular cholesterol transport and homeostasis. This test has been considered the gold standard method for diagnosing NP-C because it establishes the biochemical phenotype of the disease and provides useful functional evidence of the pathogenicity of novel gene mutations [4, 7–10]. However, recent progress in gene mutation analysis, the lack of correlation between some causal mutations and ‘variant’ filipin staining patterns, and the fact that the filipin test is time consuming and expensive, have led to this gold standard being challenged [8, 11]. Most patients with NP-C (80–85%) show a ‘classical’ pattern of cholesterol storage featuring numerous, strongly fluorescent perinuclear vesicles. However, some patients display a ‘variant’ biochemical phenotype that features a less distinct, more variable pattern [9, 10].

The establishment of reliable biomarkers for the presence and progression of NP-C represents an important goal. Chitotriosidase (ChT) is a human plasma chitinase enzyme that shows markedly elevated activity in a variety of lysosomal storage disorders [12, 13], High plasma levels reflect gradual intralysosomal accumulation of the enzyme in lipid-loaded macrophages, which secrete it [14]. Plasma ChT is widely used in Gaucher disease (GD) to monitor treatment response to enzyme replacement therapy (ERT) [12]. Ries et al. reported that patients with NP-C showed a mean plasma ChT activity of 856 ± 721 nmol/mL*h compared with 55 ± 35.6 nmol/mL*h in individuals with miscellaneous other diseases, and 13,761 ± 10,348 nmol/mL*h in patients with GD [15]. A ChT activity >200 nmol/mL*h is considered pathological [15]. However, 3–6% of individuals with European ancestry have the c.1049_1072dup24 polymorphism of the ChT gene (*CHIT1*), which leads to a complete lack of ChT activity [16, 17].

Chemokine (C–C motif) ligand 18 (CCL18)/pulmonary and activation-regulated chemokine (PARC) is produced mainly by monocytes/macrophages that constitutively express it only at low levels in normal circumstances, but its production can be up-regulated in these cells by macrophage activators [18]. Plasma CCL18 levels have been reported to increase 29-fold in symptomatic GD patients [19] leading to its use as a further disease biomarker [20]. Pineda et al. observed that patients with early- or late-infantile onset NP-C had higher plasma CCL18/PARC activities compared with juvenile-onset or asymptomatic patients, and proposed CCL18/PARC as an alternative marker in NP-C patients with ChT deficiency [20, 21].

Levels of several cholesterol oxidation products (oxysterols) have recently been proposed as sensitive and specific markers for NP-C screening and/or diagnosis. Increased plasma 7-ketocholesterol (7-KC) levels have been reported in NP-C patients and in an NP-C mouse model [7, 22]. Further, Porter et al. have described an oxysterol profile specific for NP-C that correlates with the age of disease onset as well as disease severity [23].

In the following study, we evaluated whether plasma ChT activity and CCL18/PARC concentration might complement clinical assessments for the identification of patients who should undergo further, specific testing for NP-C. We also compared these biomarker analyses with NP-C SI scores and with findings from plasma oxysterol measurements.

## Methods

### Patients and study design

Patients from 51 Spanish medical centres specializing in neurodegenerative disorders who had two or more symptoms typically seen in NP-C were considered as possibly having the disease, and were enrolled into this prospective observational study combined with a retrospective determination of NP-C SI scores following reference to a questionnaire with a list of symptoms and clinical data of interest (see Additional file 1). Patients presenting with at least two of the following symptoms were included in the study: bipolar disorder; schizophrenia; depression; one other clinical symptom. Medical chart data relating to demographics, diagnosis, and recorded disease characteristics were obtained from referring physicians.

The study comprised two observation periods, during which different diagnostic approaches were followed. In Period 1, which ran from January 2010 to April 2012, patients with two or more clinical NP-C signs and symptoms were considered as having ‘suspected NP-C’. *NPC1*/*NPC2* sequencing, ChT and sphingomyelinase activities, and CCL18/PARC concentration were assessed in all patients. If either one or two *NPC1* or *NPC2* mutations were identified, filipin staining and oxysterol measurements were also performed, where possible. In Period 2, which ran from May 2012 to April 2014, ChT activity, CCL18/PARC concentration and NP-C SI scores were analyzed in all patients, and *NPC1*/*NPC2* sequencing was performed only in those who had elevated ChT and CCL18/PARC values (greater than mean + two standard deviations [SD] versus control values), *and/or* an NP-C SI score ≥70. Filipin staining and/or plasma oxysterol measurements were performed, where possible, in all patients with either one or two *NPC1* or *NPC2* mutations.

### Genetic analysis

DNA was isolated from EDTA blood samples using standard methods. The promoter and 25 exons of *NPC1*, and respective exon–intron boundaries, were amplified simultaneously in 24 PCR reactions using oligonucleotide primers developed in-house at the University of Zaragoza. The promoter, coding regions of the five exons of *NPC2* and respective exon–intron boundaries were amplified in a single multiplex reaction with linker-tailed primers. Amplification products were joined into one DNA fragment using universal external primers, and the resulting amplicons were purified using the Illustra™ ExoStar™ 1-Step system (GE Healthcare, UK), followed by 5′ to 3′ sequencing in an ABI 3500XL DNA analyzer (Applied Biosystems, USA).

To analyze splicing variants, total RNA was isolated from cultured fibroblasts (1 × 10^6^ cells/column) using the RNeasy Mini Kit (Qiagen, USA), and genomic DNA was removed using the RNase-Free DNase Set (Qiagen). RNA (200 ng for each reaction) was used for cDNA synthesis with random hexamer primers using RevertAid H (Qiagen) minus first strand cDNA synthesis. Two fragments, one from c.227 to c.976 and another from c.1793 to c.2359, were amplified by PCR, purified using the Illustra™ ExoStar™ 1-Step system (GE Healthcare), followed by 5′ to 3′ sequencing in an ABI 3500XL DNA analyzer (Applied Biosystems). All primer sequences used for PCR are available upon request.

Patients with only one *NPC1* mutation were further analyzed by Multiplex Ligation-dependent Probe Amplification (SALSA^®^ MLPA^®^ P193 *NPC1* version A2; MRC Holland, Netherlands) in order to find any rearrangements, with data normalized *versus* three healthy controls.

A number of software databases (PolyPhen-2 [24], SIFT [25] and MutationTaster [26]) were used to evaluate the pathogenicity of newly identified genetic variants that implied non-synonymous changes. The effect of variants in potential splicing sites was predicted using NetGene2 and NNSplice by analyzing the structure of donor and acceptor sites with a separate neural network recognizer for each site [27, 28]. Common polymorphisms were excluded, and gene variations were compared with databases of the National Center for Biotechnology Information (NCBI; http://www.ncbi.nlm.nih.gov/), Ensembl (http://www.ensembl.org/), the 1000 Genomes project (http://www.1000genomes.org/), and the Exome Variant Server (http://evs.gs.washington.edu/EVS/). All mutations were described according to the latest Human Genome Variation Society (HGVS) recommendations (http://www.hgvs.org/mutnomen).

### Chitotriosidase activity and CCL18/PARC concentration

Plasma for biomarker measurements was separated from EDTA blood samples. Plasma ChT activity was measured using the fluorogenic substrate 4MU-chitotrioside (Sigma Chemical Co., USA), as described previously [12]. Genotyping for the 24-bp insertion in exon 10 of the *CHIT1* gene (c.1049_1072dup24) was performed as described by Irun et al. [16]. Given that ChT activity is roughly half of normal or zero in patients who are heterozygous and homozygous for the c.1049_1072dup24 *CHIT1* mutation, respectively, ChT levels were multiplied by two in heterozygous patients. The concentration of the chemokine CCL18/PARC was analyzed by enzyme-linked immunosorbent assay (ELISA; R&D Systems Europe, Ltd, UK) as described elsewhere [19]. Mean ± SD control values determined in 36 patients who did not have lysosomal disorders were: 46.1 ± 30.2 nmol/mL/h for ChT activity, and 52.5 ± 30.3 ng/mL for CCL18/PARC concentration.

### NP-C suspicion index

Individuals >4 years old were assessed using the NP-C SI as described in detail elsewhere (http://www.npc-si.com) [6]. The NP-C SI was published in 2012, but it was considered useful in the context of the current study and was therefore applied retrospectively in Period 1 and prospectively in Period 2.

### Filipin staining

Normal and NP-C fibroblasts were cultured using standard laboratory methods, but with some adaptations (see Additional file 2). Only homogeneous confluent cell monolayers grown over 3–6 passages, and covering an area of 25 cm^2^, were studied. Fluorescent staining of lysosomal cholesterol in fibroblast cultures was performed based on a well-known cytochemical method in low-density lipoprotein (LDL)-challenged cells, and perinuclear cholesterol accumulation was assessed as described previously [9].

### Oxysterol analysis

The concentration of 7-KC was measured in all patients with available plasma samples and at least one identified *NPC1* mutation. Extraction of 7-KC from plasma samples was conducted according to the method described by Lin et al. [22], and 7-KC was quantified using liquid chromatography-tandem mass spectrometry (LC–MS/MS) based on a slightly modified version of the method described by Baila-Rueda et al. [29]. Briefly, a calibration curve for 7-KC showed a correlation coefficient of 0.995, with an assay linear range of 2–800 ng/mL. The lower limits of detection (LOD) and quantitation were 1 and 2 ng/mL, respectively. Intra-day and inter-day variations were <5 and <11%, respectively. Mean ± SD control values determined in 36 patients who did not have lysosomal disorders were 15.99 ± 14.67 ng/mL.

### Family studies

Genetic analyses were applied in all available first-degree relatives of index cases possessing two *NP*-*C* gene mutations in order to establish whether mutations were located in the same allele or in different alleles, and to identify other NP-C patients.

### Statistical methods

As this was an observational study, data analyses were descriptive in nature.

## Results

### Patients and diagnoses

**Fig. 1:**
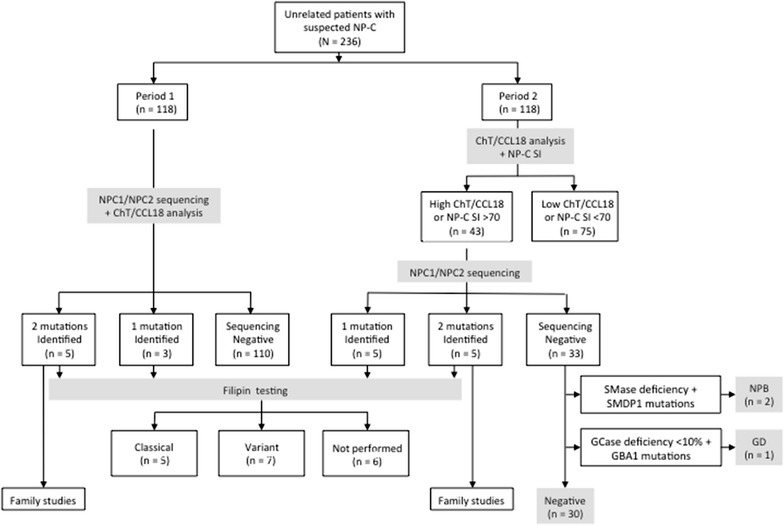
Patient flow and diagnosis

In total, 236 unrelated patients with suspected NP-C were included in Periods 1 and 2 (Fig. 1). All 118 patients included during Period 1 underwent *NPC1/NPC2* sequencing, among whom five patients were found to be homozygous or compound heterozygotes for *NPC1* mutations (Table 1), and three were found to be carriers of single heterozygous *NPC1* mutations (Table 2).

**Table 1:** Mutational and biochemical features of NP-C patients with two *NPC1* mutations

Patient ID	Variant allele 1 amino acid	Variant allele 1 reference	Variant allele 2 amino acid	Variant allele 2 reference	CCL18/PARC ng/mL	ChT(dup24) nmol/mL.h	NP-C SI score	Filipin staining	7-KC ng/mL	Clinical form
*Period 1*										
NPC1A	p.(Gln775Pro)	[32]	p.(Asp1097Asn)	[31]	156	188 (Het)	145	ND	260	Adult
NPC2A	p.(Arg1059*)	[30]	p.(Arg1059*)	[30]	788	1045 (Neg)	NC (<4 y.o.)	Classical	650	Early infantile
NPC3A	p.(Pro1007Ala)	[36]	p.(Asn222Ser)	[34]	88	266 (Neg)	60	Variant	103	Adult
NPC4A	p.(Arg518Trp)	[35]	p.(Gly992Trp)	[36]	151	244 (Neg)	195	ND	213	Adult
NPC5A	p.(Trp942Cys)	[30]	p.(Arg1173Gly)	New	266	156 (Het)	95	ND	178	Adult
*Period 2*										
NPC6A	p.(Arg372Trp)	[30]	p.(Thr1036Met)	[2]	466	415 (Neg)	20	ND	238	Adult
NPC7A	p.(Glu1188 fs*54)	[30]	p.(Thr375Ala)	New	265	75 (Neg)	200	ND	398	Adult
NPC8A	p.(Ile1061Thr)	[17]	p.(Ile1061Thr)	[17]	1137	1477 (Neg)	NC (<4 y.o.)	ND	514	Early infantile
NPC9A	p.(Cys177Tyr)	[35]	p.(Val664Met)	[34]	516	614 (Neg)	190	Classical	193	Adult
NPC10A	p.(Leu107Cfs*5)	[32]	p.(Glu61 + ?_Asp211 + ?)dup	New	1048	812 (Het)	NC (<4 y.o.)	Classical	761	Early infantile

Mutations were described according to the latest HGVS recommendations (http://www.hgvs.org/mutnomen); *ChT* chitotriosidase (ChT activity of heterozygous individuals [dup24] was multiplied by two). *NC* not conducted (patient < 4 years old), *ND* no data available, *Neg* negative, *7*-*KC* 7-ketocholesterol, *LOD* limit of detection (= 2 ng/mL)

**Table 2:** Mutational and biochemical features of ‘NP-C uncertain’ patients with only one *NPC1* mutation

Patient ID	Variant allele 1 amino acid	Variant allele 1 reference	CCL18/PARC ng/mL	ChT (dup24) nmol/mL.h	NP-C SI score	Filipin staining	MLPA	7-KC ng/mL	Clinical form
*Period 1*									
NPC1B	p.(Leu846Pro)	New	233	109 (Neg)	55	Variant	Negative	134	Adult
NPC2B	p.(Phe1221Sfs*20)	[30]	123	53 (Neg)	60	Classical	Negative	34	Adult
NPC3B	p.(Gln775Pro)	[32]	198	167 (Neg)	55	Classical	Negative	<2	Adult
*Period 2*									
NPC4B	p.(Arg1274Trp)	New	ND	ND	NC (<4 y.o.)	Variant	Negative	ND	Early infantile
NPC5B	p.(Glu451Lys)	[36]	550	771 (Neg)	NC (<4 y.o.)	Variant	Negative	258	Early infantile
NPC6B	p.(Asn222Ser)	[34]	138	59 (Neg)	NC (<4 y.o.)	Variant	Negative	150	Late infantile
NPC7B	p.(Gln775Pro)	New	416	132 (Neg)	101	Variant	Negative	125	Adult
NPC8B	p.(Gln775Pro)	[32]	39	24 (Neg)	120	Variant	Negative	19	Juvenile

Mutations were described according to the latest HGVS recommendations (http://www.hgvs.org/mutnomen); *ChT* chitotriosidase (ChT activity of heterozygous individuals [dup24] multiplied by two), *NC* not conducted (patient <4 years old), *ND* no data available, *Neg* negative, *7*-*KC* 7-ketocholesterol, *LOD* limit of detection (=2 ng/mL)

In Period 2, 118 patients were assessed for biomarkers (ChT and CCL18/PARC). But based on biomarkers results, only 43 out of 118 patients in Period 2 underwent genetic testing. Five of the 118 patients enrolled during Period 2 were homozygous or compound heterozygotes for *NPC1* mutations (Table 1), and a further five were carriers of one *NPC1* allele variant (Table 2). Sphingomyelinase deficiency was detected in two patients who were subsequently diagnosed with Niemann-Pick disease type B based on *SMPD1* gene mutation analysis. Another patient showed acid glucosidase deficiency (with <10% of normal activity), and was diagnosed with GD after identification of two *GBA* mutations.

### ChT and CCL18/PARC analyses

The mean ± SD plasma CCL18/PARC concentration and ChT activity in the five patients from Period 1 with an identified genetic cause for NP-C were 289 ± 286 ng/mL (range 88–788 ng/mL) and 380 ± 374 nmol/mL*h (range 156–1045 nmol/mL*h), respectively. Patients with one mutation had substantially lower values: 185 ± 56.3 ng/mL (range 123–233 ng/mL) and 110 ± 56.1 nmol/mL*h (range 53–167 nmol/mL*h), respectively. Excluding patient NPC4B who had no available data, all patients with *NPC1* mutations showed CCL18/PARC concentrations and/or corrected ChT activities that were at least two SDs greater than control values.

In Period 2, 43/118 (36%) patients had CCL18/PARC concentrations and/or corrected ChT activities at least two SDs greater than control values (i.e., CCL18/PARC >115 ng/mL and/or ChT >150 nmol/mL*h) [16], or normal biomarker levels but an NP-C SI score >70. In this Period, 18 patients showed elevated levels of both ChT and CCL18/PARC, six only showed elevated ChT activity, and 14 had elevated CCL18/PARC concentrations. Five patients had only NP-C SI ≥70, and 13/118 subjects from Period 2 had NP-C SI ≥70. *NPC1* and *NPC2* mutation analyses were therefore conducted in 43 patients in this group.

Analysis for the c.(1049_1072) dup24 *CHIT1* genotype identified four and three homozygous patients in Periods 1 and 2, respectively, and five and 10 heterozygous patients in Periods 1 and 2, respectively. The homozygous patients were not excluded from the study because it was still possible to analyze CCL18/PARC concentration and NP-C SI scores.

### NP-C suspicion index analysis

Overall, 79 (33%) of all enrolled patients had an NP-C SI risk prediction score ≥70 following clinical screening, 17 (7.1%) of whom were children <4 years old. Six out of 10 of the NP-C patients who had two *NPC1* mutations had a high suspicion of NP-C (risk prediction score ≥70) (Table 1). The remaining patients were either <4 years old or had an NP-C SI <70. Among patients in the single-*NPC1* mutation group, of which four were children aged <4 years, only one patient had an NP-C SI ≥70 (Table 2).

### Filipin staining

Skin biopsies were performed in all patients who consented to them, and diagnoses of NP-C were confirmed based on abnormal filipin staining in these cases (Table 1). Six patients with a single *NPC1* mutation showed a variant filipin staining pattern, while two showed a classical staining pattern (Table 2).

### Oxysterol analysis

Plasma 7-KC concentrations were analyzed in 17 NP-C patients and 21 relatives. The mean ± SD 7-KC level among all patients with two *NPC1* mutations was 350.8 ± 221.8 ng/mL (range 103–761 ng/mL), and all of these patients had plasma 7-KC concentrations higher than the optimal control cut-off value (102.8 ng/mL) in our laboratory (Table 3). Among patients with one mutation, the mean ± SD 7-KC concentration was also raised (194 ± 265.9 ng/mL [range <2–761 ng/mL]), although only 4/7 of these patients, all of whom had positive classical or variant filipin staining results, had plasma 7-KC concentrations higher than the cut-off value.

**Table 3:** Patient demographics and diagnostic features among all study patients

	NP-C positive (two *NPC1* mutations) (n = 10)	NP-C uncertain (one *NPC1* mutation) (n = 8)	NP-C negative (n = 218)
*Age (years)*			
Mean ± SD	28 ± 21	28 ± 28	44 ± 22
Median (range)	35 (1.4–62)	21 (0.8–68)	46 (0.03–83)
*Gender* *(n)*			
Male	4	6	126
Female	6	2	92
*NP*-*C SI* *(points)*			
Mean ± SD	129 ± 72.1	78.2 ± 27.1	58.9 ± 39.2
Median (range)	145 (20–200)	69.1 (55–120)	55 (5–245)
*ChT activity* *(nmol/mL/h)*			
Mean ± SD	553 ± 479	187 ± 262	122 ± 423
Median (range)	255 (75–1477)	109 (24–771)	53 (11–5149)
*CCL18 conc.* *(ng/mL)*			
Mean ± SD	481 ± 370	242 ± 179	95 ± 178
Median (range)	266 (88–1137)	198 (39–550)	(11–1513)
*7*-*KC* *(ng/mL)*			
Mean ± SD	351 ± 222	103 ± 91.20	ND
Median (range)	249 (103–761)	198 (<2–761)	
*Filipin staining (n)*			
Classical	3	2	ND
Variant	1	6	
No data	6	0	

All patients, including those from both Periods 1 and 2. Data expressed as mean ± SD, median and range (minimum–maximum). Normal biomarker values calculated in 36 patients without lysosomal disorders were: 46.1 ± 30.2 nmol/mL/h for ChT activity; 52.5 ± 30.3 ng/mL for CCL18/PARC concentration; and 15.99 ± 14.67 ng/mL for 7-KC concentration. *ND* no data available

### Clinical manifestations

Clinical manifestations are summarized in Table 4. The most prevalent manifestations in patients with a confirmed diagnosis of NP-C were neurological signs, including: ataxia, VSGP, dementia, dystonia and/or dysarthria. Psychiatric signs were the second most frequent class of manifestation, and included pre-senile cognitive decline/dementia, psychotic symptoms, and depression/bipolar disorders. Unexplained splenomegaly with or without concurrent hepatomegaly were present in 5/10 patients (50%) with a confirmed diagnosis.

**Table 4:** Clinical disease characteristics of all patients

	NP-C positive (two *NPC1* mutations) (n = 10)	NP-C uncertain (one *NPC1* mutation) (n = 8)	NP-C negative (n = 218)
*Neurological symptoms, n (%)*			
Vertical supranuclear gaze palsy (VSGP)	3 (30%)	3 (37.5%)	58 (26.6%)
Gelastic cataplexy	0 (0%)	0 (0%)	7 (3.2%)
Ataxia, clumsiness or frequent falls	5 (50%)	3 (37.5%)	89 (40.8%)
Dysarthria and/or dysphagia	2 (20%)	3 (37.5%)	31 ((14.2%)
Dystonia	1 (10%)	1 (12.5%)	55 (25.2%)
*Psychiatric symptoms present, n (%)*			
Pre-senile cognitive decline or dementia	2 (20%)	1 (12.5%)	76 (34.9%)
Psychotic symptoms (schizophrenia)	1 (10%)	0 (0%)	19 (8.7%)
Depression	3 (30%)	2 (25%)	49 (22.5%)
Bipolar disorders	1 (10%)	1 (12.5%)	12 (5.5%)
*Visceral symptoms, n (%)*			
Unexplained neonatal jaundice or cholestasis	1 (10%)	2 (25%)	12 (5.5%)
Unexplained splenomegaly ± hepatomegaly	6 (60%)	3 (37.5%)	15 (6.9%)
Hydrops fetalis or fetal ascites	0 (0%)	0 (0%)	1 (0.5%)

Including those from both Periods 1 and 2

### NPC1 mutations

Patients diagnosed with NP-C in this cohort showed wide heterogeneity of *NPC1* variants. In total, eight new *NPC1* variants were detected: four missense (p.(Thr375Ala), p.(Leu846Pro), p.(Arg1173Gly), p.(Arg1274Trp)), two different tentative splicing variants at intron 12 (c.(1947 + 10G > A)) and exon 5 (c.(612C > T), p.(Thr204Thr)), one deletion (c.318_318delC) that produces a frameshift change, p.(L107CfsX5), and one rearrangement (c.(280 + ?_c.630 + ?) dup (p.Glu61 + _Asp211 + ?)dup)) that leads to a duplication of exons 4 and 5. *In silico* analysis with Polyphen2, SIFT and MutationTaster software indicated that these changes could affect protein function (Table 5). We also found 17 rare *NPC1* variants previously associated with NP-C. A further 17 common *NPC1* variants or polymorphisms were also observed, including: c.(−22A > C), p.(Tyr129Tyr), p.(His215Arg), p.(Pro237Ser), p.(Ser322Ser), p.(Asn490Thr), p.(Met642Ile), p.(Ile858Val), p.(Asn931Lys), c.(1947 + 10G > C), c.(1947 + 14G > T), c.(2086 + 8G > C), c.(2911 + 28T > C), c.(3246 + 46C > T), c.(3591 + 35C > T), c.(3754 + 34A > G), and p.(Arg1266Gln). Five *NPC2* polymorphisms were detected (p.(Gly52Gly), p.(Ser67Pro), p.(Pro86Leu), p.(Ser121Ala) and c.(441 + 437T > C)), but no allele mutations were identified.

**Table 5:** In silico mutation predictions

Mutation	PolyPhen-2	SIFT	Mutation taster
p.(Leu107CfsX5)	NA	NA	Disease-causing
p.(Thr375Ala)	PrD (0.997)	Damaging (0.010)	Disease-causing
p.(Leu846Pro)	PrD (0.995)	Damaging (0.001)	Disease-causing
p.(Arg1173Gly)	PrD (0.999)	Damaging (0.007)	Disease-causing
p.(Arg1274Trp)	PrD (0.986)	Damaging (0.004)	Disease-causing
p.(Thr204Thr)	NA	Tolerated (0.703)	Disease-causing
c.(1947 + 10G > A)	NA	NA	Polymorphism

PolyPhen-2 score ranges from 0 to 1, with mutations qualitatively appraised as PrD (probably damaging), PoD (possibly damaging), or B (benign). MutationTaster analyses the probability that a variant will be disease-causing or is a polymorphism. SIFT scores range from 0 to 1: the amino acid substitution is predicted as damaging if the score is ≤ 0.05, and tolerated if the score is > 0.05. *NA* not possible to analyze

Two variants of uncertain significance were detected (c.(1947 + 10G > A) and c.(612C > T)). Complementary DNA sequencing was conducted in fibroblast DNA obtained from patient NPC10 to better classify these two variants, and confirmed the presence of heterozygous mutations (p.(Leu107CysfsX5) and c.(612C > T) [p.Thr204Thr]). We also identified the c.(280 + ?_c.630 + ?)dup variant. The c.(1947 + 10G > A) mutation was analyzed, and a splicing effect has not been observed.

### Family studies

Forty-nine relatives were available for genetic analysis, and 22 were identified as carriers of at least one *NPC1* mutation. Three relatives were identified as compound heterozygotes for *NPC1* mutations: p.(Pro1007Ala)-p.(Asn222Ser); p.(Trp942Cys)-p.(Arg1173Gly); and p.(Glu1188 fs*54)-p.(Thr375Ala). Further examination of these three individuals revealed clinical symptoms of NP-C, and they were subsequently diagnosed with NP-C.

## Discussion

Due to the variability of age at onset and presentation in NP-C it is crucial to determine which diagnostic strategy might best help identify affected patients among suspected cases. We evaluated two different diagnostic pathways incorporating two well characterized biomarkers that have previously been used to monitor lysosomal storage disease progression (ChT activity and CCL18/PARC concentration) alongside appraisals of clinical symptoms and NP-C SI assessments.

The diagnostic approach in Period 1 was to sequence all exon and exon–intron boundaries of *NPC1* and *NPC2* in patients with suspected NP-C based on the presence of two or more typical signs/symptoms. All patients with two *NPC1* mutations, or a single *NPC1* mutation plus a positive filipin test, had elevated ChT activity and/or CCL18/PARC concentration. Ninety-eight patients without *NPC1* mutations did not show elevated ChT activity or CCL18/PARC concentration, but retrospective NP-C SI assessments showed that 42 of these patients had NP-C SI scores ≥70. Based on findings from Period 1, during period 2 we conducted *NPC1* and *NPC2* sequencing only in patients with elevated CCL18/PARC concentration and/or ChT activity, or with an NP-C SI ≥70 for all individuals >4 years old. During this second period, five patients with homozygous or compound-heterozygous *NPC1* mutations were successfully identified. Five carrier heterozygote patients with single *NPC1* variants and positive filipin test findings (classical or variant biochemical phenotypes) were also detected.

We observed the same NP-C detection rate (5/118; 4.2%) using the criteria of study Period 1 (i.e., *NPC1* and *NPC2* sequencing in all cases of suspected NP-C) as we did using the criteria of study Period 2 [i.e., *NPC1* and *NPC2* sequencing only when the ChT activity and/or CCL18/PARC concentration were elevated and/or NP-C SI was ≥70; 5/118 cases (4.2%)]. These findings suggest that the measurement of one or both of plasma ChT activity and CCL18 concentration in conjunction with assessment of NP-C SI score in patients with suspected NP-C allows better targeting of in-depth confirmatory laboratory tests. A reduced number of patients were referred for confirmatory molecular-genetic testing, enabling decreases in both the time and costs associated with diagnosing NP-C.

Considering findings from both study periods together, although the majority of NP-C patients with two *NPC1* mutations had elevated levels of both ChT and CCL18/PARC, two subjects had a clear genetic diagnosis of NP-C but showed raised levels of only one of these biomarkers.

Across the two periods in the current study, we identified a total of six novel *NPC1* variants and 17 *NPC1* mutations that have been described previously [2, 17, 30–36]. No causal *NPC2* mutations were detected. Eight of the patients examined in this study only had a single *NPC1* mutation, and it is considered highly likely that all of these patients are true cases of NP-C based on their clinical presentation, filipin staining findings, and plasma ChT, CCL18 and 7-KC values. It is possible that these patients also had a second point mutation that was not detectable with our sequencing and MLPA methodology. Alternatively, these mutations could have resulted from whole-gene deletions, intron sequence variants or mutations in other genes that could modify intra-lysosomal cholesterol transport. Full-gene sequencing is possible using next-generation methods, but this is likely to identify variants that are difficult to interpret.

We observed that patients with homozygous or compound-heterozygous *NPC1* mutations, as well as carriers of single *NPC1* mutations, had elevated ChT activity and CCL18/PARC concentration, although levels of these biomarkers were higher in NP-C positive patients than in ‘NP-C uncertain’ individuals. The existence of *CHIT1* polymorphisms associated with reduced ChT activity limits the usefulness of ChT as a biomarker in some patients [16]. Nonetheless, the combined analysis of both ChT and CCL18/PARC levels allowed the identification of four additional patients with lysosomal storage diseases: three patients with Niemann-Pick disease type B and one with GD.

The NP-C SI, which was developed in 2012, was applied retrospectively to individuals assessed during Period 1 and prospectively in those assessed during Period 2 in order to gain a complete set of diagnostic data. This clinical tool has shown promise in aiding the diagnosis of NP-C in previous studies [6]. However, we observed high prediction scores (≥70) in 79 of all included patients >4 years old in the current cohort, in which only six patients had a confirmed diagnosis of NP-C. While several patients in this cohort had high NP-C SI scores as well as clinical signs associated with NP-C (VSGP and splenomegaly), genetic analyses did not reveal any *NPC1* or *NPC2* mutations. Conversely, the NP-C SI cut-off value of 70, recommended as an indicator for additional diagnostic tests, could have led to non-detection of NP-C in two patients who had scores of 60 and 20. The measurement of plasma ChT activity and CCL18/PARC concentration as well as NP-C SI assessments allowed us to confirm a total of eight NP-C diagnoses. In addition, two patients in whom the NP-C SI was not applicable due to their age (<4 years) were detected based on biomarker measurements alone.

It should be borne in mind that elevations in CCL18/PARC concentration are not exclusively caused by lysosomal disorders. CCL18/PARC is a circulating chemokine that plays a role in injury healing, physiological homing of mononuclear blood cells, and inflammatory responses [37]. Indeed, some studies have indicated that CCL18/PARC is expressed in atherosclerotic plaques, and represents an independent risk predictor of short-term mortality in patients with acute coronary syndromes [38]. The lack of correlation between CCL18/PARC levels and NP-C SI scores in this study might therefore be due to the presence of other concomitant pathologies such as cardiovascular disease, which are not considered in the NP-C SI.

The filipin test was performed in as many patients as possible in this cohort, and all tested patients with one or two *NPC1* mutations showed a classical or variant filipin staining pattern, respectively. However, it is interesting to note that not all carriers of single *NPC1* mutations who had a positive filipin result exhibited elevated plasma 7-KC concentrations, whereas all patients with two *NPC1* mutations had 7-KC levels above the control cut-off value in our laboratory. It is also notable that family studies addressing ChT/CCL18/7-KC biomarkers in conjunction with the NP-C SI, filipin testing and subsequent gene sequencing led to the identification of a further three NP-C patients, and enabled the validation of two new causal *NPC1* variants (p.(Arg1173Gly) and p.(Thr375Ala)).

This investigation has several limitations and strengths that should be taken into account in interpreting the reported findings. Although the inclusion of patients with two typical NP-C symptoms might be considered a bias for having a high NP-C SI score, it should be noted that this study was started in 2010, and the symptoms required for inclusion in this work were specified before the NP-C SI was developed and published in 2012 [6]. Such a bias was therefore not possible. Secondly, we did not sequence all individuals with suspected NP-C who had plasma ChT activity or CCL18/PARC concentration less than the mean control value (plus two SDs). However, we did conduct NP-C gene sequencing and MLPA in all individuals with an NP-C SI ≥70 or who were <4 years old. Thirdly, the sequencing techniques that we employed only covered the coding regions of the *NPC1* and *NPC2* genes and their intron–exon boundaries, and therefore might not have detected regulatory and deep intronic splicing mutations. It is also possible that some novel mutant NP-C gene alleles might not yet have been characterized, and therefore may be present (but go undetected) among ‘NP-C uncertain’ or even ‘NP-C negative’ individuals. Finally, the genetic diagnosis of NP-C requires the identification of clearly pathogenic mutations, while many families have ‘private’ sequence variants that have not yet been reported/published. While in silico protein and splicing prediction tools can be employed to assist in assigning pathogenicity to sequence variants resulting from missense or intronic changes, decisions regarding the pathogenicity of novel, private mutations are generally very difficult to make.

## Conclusion

We conclude that plasma ChT activity and CCL18/PARC concentration measurements in patients with suspected NP-C based on their clinical symptomatology can help pave the way to conducting specific, confirmatory laboratory tests. This approach can be very useful for laboratories that do not have mass spectrometry facilities and therefore they cannot use other NP-C biomarkers such lyso-sphingomyelin-509 or bile acids. Patients with a plasma ChT activity and/or plasma CCL18/PARC concentration greater than two SDs beyond control values and an NP-C SI score ≥70 should undergo *NPC1* and *NPC2* gene sequencing as standard. In addition, identification of two pathogenic NP-C gene mutations that segregate in the family should be considered sufficient for at least an initial diagnosis.

## Additional files

**Additional file 1.** Patient questionnaire.

**Additional file 2.** Filipin staining methodology.

## Abbreviations

7-KC: 7-ketocholesterol
CHIT1: c.1049_1072dup24 polymorphism of the ChT gene
CCL18/PARC: chemokine (C–C motif) ligand 18/pulmonary and activation-regulated chemokine
ChT: chitotriosidase
ELISA: enzyme-linked immunosorbent assay
ERT: enzyme replacement therapy
EVS: exome variant server
GBA: glucocerebrosidase
GD: Gaucher disease
HGVS: Human Genome Variation Society
MLPA: multiplex ligationdependent probe amplification
NCBI: National Center for Biotechnology Information
LOD: limits of detection
LC–MS/MS: liquid chromatography-tandem mass spectrometry
LDL: lowdensity lipoprotein
MLPA: multiplex ligation-dependent probe amplification
NP-C: Niemann Pick disease type C
NPC1/NPC2: NP-C disease-causing genes 1 and 2
NP-C SI: NP-C suspicion index
SD: standard deviation
SMDP1: acid sphingomyelinase gene
VSGP: vertical supranuclear gaze palsy
