# Supplementary material for: Assessment of plasma chitotriosidase activity, CCL18/PARC concentration and NP-C suspicion index in the diagnosis of Niemann-Pick disease type C: a prospective observational study
Source: J Transl Med. 2017 Feb 21;15:43. doi: 10.1186/s12967-017-1146-3 (PMC5320753; doi:10.1186/s12967-017-1146-3)
Supplement: Supplementary file 1 — Additional file 1. Patient questionnaire. [file 12967_2017_1146_MOESM1_ESM.docx]

**Supplement 1.** Patient questionnaire

**PATIENT DATA** (or relative, please specify the relationship).

**Name:**

Data of birth: Place of birth: Age of onset:

Gender: M/F Weight: ___ Kg Height: ___ cm

**CLINICAL PROFILE**

**Visceral affectation**

Hepatomegaly of __ cm by ecography or physical exploration

Splenomegaly of __ cm by ecography or physical exploration

Ascites at birth: Yes No

Jaundice at birth: Yes No

**Neurological affectation**

Age of onset:

Dementia: Yes No

Depression: Yes No

Cataplexy: Yes No

Gelastic seizures: Yes No

Bipolar disorder: Yes No

Schizophrenia: Yes No

Ataxia: Yes No

Dystonia: Yes No

**Ophthalmic affectation**

Cherry stain (Exclusion criterion): Yes No

Vertical supranuclear gaze palsy: Yes No

Ophtalmoplegia supanuclear: Yes No

**Explorations**

EEG: Yes No

CT scan: Yes No

Evoked potentials: Yes No

**Biochemical analytical**

Glucose: ___ mg/dL

Creatinine: ___ mg/dL

Cholesterol: ___ mg/dL

Triglycerides: ___ mg/dL

ALT: ___ U/L

AST: ___ U/L

GGT: ___ U/L

D Bilirubin: ___ mg/dL

Alkaline phosphatase: ___ U/L

Hb: ___ g/dL

Hematocrit: ___ %

Platelets: ___ x10^9^/L

Leukocytes: ___ x10^9^/L

Vacuolated Lymohocites: ___ %
