# Supplementary material for: Assessment of plasma chitotriosidase activity, CCL18/PARC concentration and NP-C suspicion index in the diagnosis of Niemann-Pick disease type C: a prospective observational study
Source: J Transl Med. 2017 Feb 21;15:43. doi: 10.1186/s12967-017-1146-3 (PMC5320753; doi:10.1186/s12967-017-1146-3)
Supplement: Supplementary file 2 — Additional file 2. Filipin staining methodology. [file 12967_2017_1146_MOESM2_ESM.docx]

**Supplement 2.** Filipin staining methodology

Fibroblasts were seeded at a density of 90,000 cells/well on coverslips at the bottom of 12-well dishes and incubated for 24 h in Minimum Essential Media (MEM) with 10% FCS, after which they were switched to MEM/10% lipoprotein-deficient serum (LPDS) medium (Millipore Ibérica, Madrid, Spain), with incubation for 48 h to deplete cell membranes of sterols. The fibroblast preparations were then incubated for 24 h in fresh medium containing LDL (50 mg/mL) (Millipore Ibérica, Madrid, Spain) to load lysosomes with cholesterol. After 24 h, the cells were washed twice with PBS and fixed in 3% paraformaldehyde for 15 min, followed by three more PBS washes, and paraformaldehyde was quenched by 10-min incubation in 20 mM glycine–PBS. Cells were then stained with 25 µg/mL Filipin III (sigma Aldrich Quimica, Madrid, Spain) in PBS (Sigma Aldrich Quimica, Madrid, Spain) for 2 h, with the coverslips protected from light, and washed a further three times. Coverslips were mounted on microscope slides with fluorescence microscope mounting medium (Dako Diagnósticos, Sant Just Desvern, Spain). Cells were observed with the UV filter set in a Nikon Eclipse Ti epifluorescence microscope (Niko Instruments Europe, Amstelveen, the Netherlands).
